# Peyronie’s disease – outcomes of collagenase *clostridium histolyticum* injection: A systematic review

**DOI:** 10.1080/2090598X.2021.1957411

**Published:** 2021-08-16

**Authors:** Austin T. Mefford, Omer Raheem, Faysal A. Yafi, Laith M. Alzweri

**Affiliations:** a School of Medicine, University of Texas Medical Branch, Galveston, TX, USA; b Department of Urology, Tulane University School of Medicine, New Orleans, LA, USA; c Department of Urology, University of California-Irvine, Orange, CA, USA; d Division of Urology, Department of Surgery, University of Texas Medical Branch, Galveston, TX, USA

**Keywords:** Peyronie’s disease, collagenase *clostridium histolyticum*, Xiaflex, intralesional injections

## Abstract

**Objective:**

To review recent literature pertaining to collagenase clostridium histolyticum (CCh)and other intralesional (IL) therapies for the treatment of Peyronie’s disease (PD).

**Methods:**

A systematic search of literature was performed using MEDLINE and PubMed.‘Peyronie’s Disease Clostridium Histolyticum’, ‘Peyronie’s Disease Intralesional’, ‘Peyronie’sDisease Causes’, and ‘Atypical Peyronie’s Disease’ were used as query entries. Inclusion criteriarequired English text from 1980 onwards and have a full text available. Records were reviewed for study power, accuracy, and relevance to our research topic. The review was conducted using the Preferred Reporting Items for Systematic Reviews and Meta-Analyses criteria.

**Results:**

Recent literature supports the notion that CCh is the most effective IL treatment forpatients with typical and atypical PD. The capstone CCh study was the IMPRESS trial thatshowed a 34% reduction in curvature with a mean (SD) – 17.0 (14.8)° reduction with IL CCh,while men in the placebo saw an average 18.2% decrease in penile bend with a mean (SD) – 9.3 (13.6)° per person (P < 0.001). A shortened protocol for IL CCh treatment offered a 31.4%reduction in curvature, while decreasing cost and office visits, potentially increasing patientcompliance. Lastly, literature shows CCh is used most in atypical cases, with ~64.8% of patients being treated with CCh, probably because of the high efficacy and safetyprofile that it offers. Serious complications associated with CCh include urethralinjury, corporal rupture, and penile fracture.

**Conclusion:**

Since the approval of CCh by the United States Food and Drug Administration in2013, it has been a staple in the treatment of PD, and here we report the continuedsuperiority of this therapy. CCh is an effective, minimally invasive option in most PDpopulations; however, recent changes have made CCh unavailable for commercial use outside the United States, impacting many patients who have previously benefited.

## Introduction

Peyronie’s disease (PD) is a pathological condition of the penis that was first described by Francois Gigot de la Peyronie in the 18th century [1], but it was reportedly characterised earlier than that [2]. Today, PD is classified as a condition in which the tunica albuginea undergoes abnormal wound repair, resulting in a fibrotic plaque [3]. The progression of PD starts with an *acute phase* that can be characterised by pain in the erect or flaccid state and the presence of tissue deposition on the shaft of the penis. This is then followed by the *chronic phase* in which the plaque has become fibrous and usually calcified, making it longstanding [4]. The plaque is rigid and does not adapt to increased blood flow during erections,which can result in pain, deformity, and sexual dysfunction [5]. Unlike routine processes, the scar resulting from immunological intervention does not resolve or remodel after the acute inflammation has been terminated, thus presenting the challenge of removing the plaque to restore normal penile appearance and functioning.

The understanding of PD has paradoxically progressed immensely, as we now know much more about the disease than ever before; however, the exact aetiology remains a mystery. One of the first studies of its kind implicated certain human leucocyte antigen (HLA) genes showing autoimmune dysregulation [6]. Other studies have shown PD to have positive correlations between uricaemia, urethritis, and Dupuytren’s contracture [7]. Although there is much evidence to support PD is a multifaceted pathology, the most acknowledged aetiology involves some form of penile trauma as the catalyst to an immunological cascade [8]. Furthermore, there does seem to be genetic alterations in genes coding for fibrosis, leucocyte chemotaxis, and inflammatory cell proliferation that can predispose one to developing PD [9,10]. However, it should be noted that some studies have been unable to link PD to a prior trauma; indeed, in a study of 150 men who suffered surgical penile fracture, none went on to develop PD [11]. The link between the genetic component and the physical trauma remains a mystery. Do the implicated mutations increase the susceptibility of the penis to experience trauma? Or does the trauma initiate an aberrant wound repair pathway that is a sequela of genetic malfunction? This could be an area of active research.

There have been a number of proposed therapies for PD. Oral medications such as potassium para-aminobenzoate, vitamin E, colchicine, acetyl L-carnitine, and tamoxifen citrate have all been studied and were not found to provide benefit to patients with PD [12–16]. But even the optimists who believe in the therapeutic properties of some of these oral medications must admit that improvement in penile curvature and function is minimal at best [17]. Oral therapies are not recommended by the AUA for the treatment of PD. The most effective way to combat the disease is by surgical intervention. Tunical plication, grafting, and penile prosthesis implantation are the most invasive procedures performed to alleviate penile curvature; these involve open procedures that allow surgeons to manually straighten the penis via three different methods [4,18,19]. The other, and less aggressive option involves intralesional (IL) injection directly into the plaque in hope to eradicate PD symptoms. Verapamil, interferon (INF) alpha-2b, hyaluronic acid (HA), and CCh (Xiaflex®, Endo Pharmaceuticals, Dublin, Ireland), are currently the most popular IL therapies [20]. The present review focusses on the outcomes of CCh injection in comparison to alternative IL therapies.

## Methods

**Figure 1.**
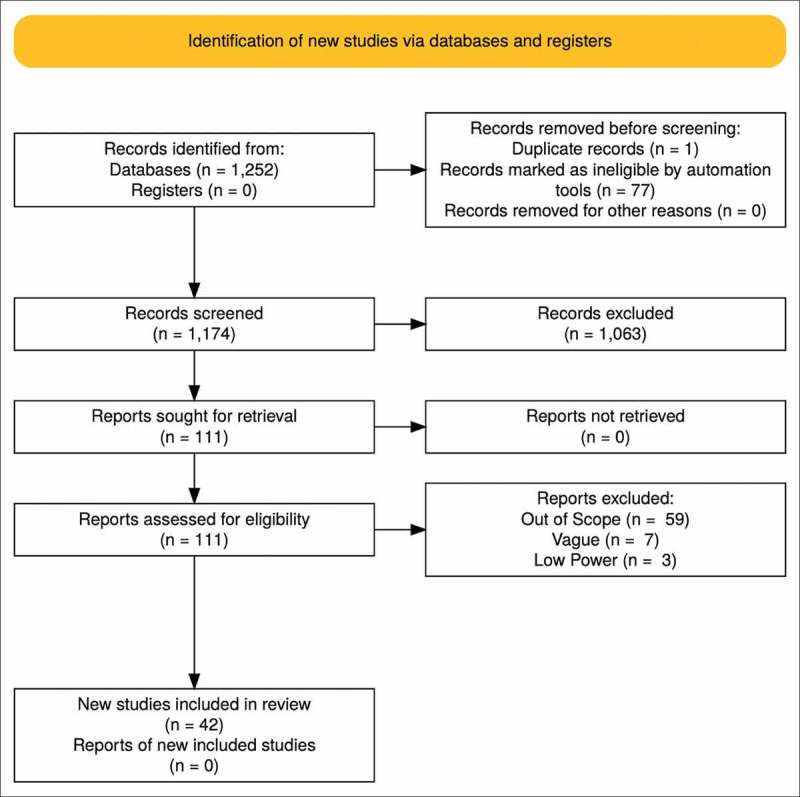
Flow chart of study selection according to the PRISMA guidelines

To approach the topic of PD and associated therapies, MEDLINE and PubMed were used to assist in data collection. We used the queries ‘Peyronie’s Disease *Clostridium Histolyticum*’, ‘Peyronie’s Disease Intralesional’, ‘Peyronie’s Disease Causes’, and ‘Atypical Peyronie’s Disease’ to gather data related to CCh; and other queries such as ‘Peyronie’s Disease Verapamil’, ‘Peyronie’s Disease Interferon Alpha’, and ‘Peyronie’s Disease Hyaluronic Acid’ for data regarding non-CCh IL therapies. The results were then filtered to exclude duplicates and include only full-text English manuscripts that were more recent than 1980. The remaining records were reviewed and screened further for rigor, specificity, and relevancy to our research interest. Of the data that passed the screening criteria, a preference was placed on high-impact, recent literature to be included in the systematic review. The research methodology followed the Preferred Reporting Items for Systematic Reviews and Meta-Analyses (PRISMA) protocol and is shown in Figure 1.

## Results

After the preliminary search of the literature, 1252 records were identified, with one duplicate removed and 77 marked as ineligible by automation tools. After the initial screen of the remaining 1174 records, 1063 were excluded due to not matching the inclusion criteria outline above. After screening and exclusions from the initial search of the literature, 111 reports remained and were then subjected to review for study power, accuracy, and relevance to our research topic. Of these full-text articles assessed for eligibility, 59 were excluded for being out of scope, seven were excluded for being too vague, and three were omitted for low power analyses. The remaining 42 articles were selected as eligible for the present review. Tables 1 [21–28] and 2 [29–35] summarise the highest impact and most clinically relevant studies to our research topicTables 2.

**Table 1.** Relevant studies utilising verapamil, INF alpha-2B and HA for the treatment of PD

Study	Design	*N*	Therapy	Objective	Duration	Outcome
Levine et al. [21]	Non-randomised, prospective, non-placebo controlled	14	IL 10 mg verapamil	Explore effects of verapamil on PD plaques	Biweekly injections for 6 months	Decreased plaque volume of >50% in 30% of patients; 83% of patients saw plaque-related changes in sexual function halt
Levine et al. [22]	Non-randomised, prospective, non-placebo controlled	46	IL 10 mg verapamil diluted to 10 mL using multiple puncture technique	Explore effects of verapamil on PD plaques using larger sample	Injections every 2 weeks for 12 total injections	54% of patients decreased penile curvature, 11% saw increase, and 34% had no change
Sadagopan et al [23]	Meta-analysis	390	–	Determine statistical outcomes of verapamil injections in patients with PD	–	IL verapamil improved sexual function (*P* < 0.001) and penile curvature (*P* < 0.005). Effect on plaque size was not significant
Wegner et al [24]	Non-randomised, prospective, non-placebo controlled	25	5 IL injections of 1 × 10^6^ IU of INF alpha-2b	Examine efficacy of IL INF therapy in patients with PD	1 injection/week for 6 months	Improvement was seen in non-calcified plaques; little to no effect on chronic plaques
Kendirci et al. [25]	Randomised, prospective, placebo-controlled, parallel	39	10 mL saline (placebo); 5 × 10^6^ IU of IL IFN alpha-2b	Compare IFN therapy to placebo on PD plaques	Injection every 2 weeks for a totally of six injections	Improvement in penile haemodynamic parameters, plaque size, and curvature; no significant change in sexual function
Zucchi et al. [26]	Prospective, single-arm, self-controlled, multicentre, pilot	65	IL 16 mg/2 mL 0.8% HA	Assess IL HA in patients with PD	1 injection/week for 10 weeks; evaluated 2 months after completion	Treatment group saw reduction in plaque size (*P* < 0.001) and curvature (*P* < 0.001)
Favilla et al [27]	Prospective, double-arm, randomised, double-blinded, multicentre	140	IL 10 mg verapamil; IL 16 mg/2 mL 0.8% HA	Comparing IL verapamil to HA in patients with PD	1 injection/week for 12 weeks	HA therapy showed reduction in penile curvature (*P* < 0.001); no change in verapamil group
Russo et al [28]	Meta-analysis	1050	–	Comparing the outcomes of IL CCh, IFN alpha-2b, verapamil, and HA on plaque properties and erectile function in patients with PD	–	CCh and IFN alpha-2b showed most reduction in curvature; HA showed best result in term of erectile function

**Table 2.** Relevant studies utilising CCh for the treatment of PD

Study	Design	*N*	Therapy	Objective	Duration	Outcome
Gelbard et al. [29]	Prospective non-placebo controlled *in vitro* analysis	6 tissue samples	Unspecified amount of CCh administered *in vitro* to PD plaques and tunica albuginea	Explore effects of CCh on fibrotic PD tissue *in vitro*	–	CCh degraded plaque and altered structure of tunica albuginea from PD tissue
Gelbard et al. [30]	Prospective randomised placebo-controlled double-blinded phase IIa trial	49	IL CCh Injection groups (0.35, 0.58, 0.81 mg) separated based on degree of curvature	IL CCh vs placebo	–	Treatment group saw decreased plaque size and penile deformity (*P* < 0.001) Established 0.58 mg as optimal dosing for IMPRESS trails
Jordan et al. [31]	Prospective non-placebo controlled single centre	25	3 IL injections of 10000 units/0.25 cm^3^ of CCh	IL CCh treatment in patients with PD	3 injections over 7–10 days with repeat treatment of 3 injections over 7–10 days 3 months later	Decreased angle of deviation (*P* < 0.001), plaque width (*P*= 0.005) and plaque length (*P* = 0.002)
Gelbard et al. [32]	2 randomised placebo controlled double blinded phase III trials	417, 415	8 IL injections of 0.58 mg CCh (2/cycle) separated by 24–72 h. After penile remodelling	IL CCh vs placebo	4 cycles of 2 injections each separated by 6 weeks	34% improvement in penile curvature with 18% improvement in placebo (*P* < 0.001).
Adbel Raheem et al. [35]	Prospective non-placebo controlled single centre	53	3 IL injection of 0.9 mg CCh (1/cycle) separated by 4 weeks. Home penile remodelling was performed in between visits	Establish a shortened protocol that is still effective for PD symptom improvement	3 cycles lasting a total of 12 weeks	96% of patients saw reduced penile curvature with a final mean of 36.9% improvement (*P* < 0.001) Established shorted IL CCh protocol
Hellstrom et al. [34]	Retrospective multi-institutional analysis	918	–	Determine statistical significance of curvature before vs after IL CCh injections	–	33% reduction in penile curvature in the 502 men who completed 4 cycles (*P* < 0.001)
Nguyen et al. [33]	Retrospective multi-institutional analysis	918	–	14.6% of men with acute PD and 85.4% with chronic PD who were all treated with IL CCh were examined for adverse events	–	No significance between CCh related adverse effects in acute vs chronic PD patients (*P* = 0.44) No significance between final degrees of curvature response to IL CCh in acute vs chronic PD (*P* = 0.09)

## Therapies

### Verapamil

Verapamil is an L-type calcium channel blocker used in the treatment of hypertension, angina, and heart failure. IL verapamil injection was brought into the spotlight after Levine et al. [21] demonstrated verapamil injection could decrease ‘plaque-associated penile narrowing’ and penile curvature. In a subsequent, and more reputable study, he showed verapamil injection reduced pain in 97% of patients, improved sexual function in 72%, and lessened the degree of curvature and deformity in 54% and 86%, respectively [22]. But other studies have shown IL verapamil to improve sexual function while not having much effect on the size of fibrous plaque [23]. It is for these mixed results and a lack of large-scale placebo-controlled studies that the United States Food and Drug Administration (FDA) has not formally approved verapamil for the treatment of PD.

### Interferon alpha-2B

The INFs are a class of cytokines that regulate immune function and injury repair. In 1995, INF alpha-2b was first studied in patients with PD and the conclusion of the study showed disappointing results of no real significance [24]. Refinements of INF alpha-2b dosing and injection intervals showed better results in decreasing plaque size and mean penile curvature in later studies [25]. When scanning the literature, many results both refute and support the use of INF alpha-2b, thus we conclude that its usefulness is inconsistent at best.

### Hyaluronic acid

Hyaluronic acid is a naturally occurring glycosaminoglycan that is used in certain conditions to reduce swelling and cytokine-mediated inflammation. The use of HA in PD is still novel and requires more investigation; however, preliminary studies have shown IL HA to reduce plaque size, lessen penile curvature, and increase sexual function in men with PD [26]. In a study where HA was compared to IL verapamil, the HA group showed a significant decrease in penile curvature (mean [SD] 4.6 [5.63]°) compared to baseline whereas verapamil did not; furthermore, men in the HA group reported a higher degree of sexual performance compared to baseline [27]. In one of the most recent studies in the literature of all IL therapies, a study determined that men who received CCh and INF alpha-2b injections showed a greater decrease in mean curvature when compared to verapamil and HA [28]. However, in the same study, they concluded that HA was most efficient in restoring erectile function when compared to other IL therapies [28].

None of the above therapies have been approved by the FDA for treatment of PD due to the lack of studies or the inconsistency of reproduceable results. However, IL CCh has been thoroughly investigated and approved by the FDA for treatment of PD, thus we will explore CCh in more depth.

### Collagenase *clostridium histolyticum*

Collagenase *clostridium histolyticum* originated in the mid-1900s when bacteria was cultured from horse Achilles tendon [36]. The scientists working with this bacteria noticed the tendon was damaged and seemingly weakened by the presence of these bacteria [36]. Upon further investigation, it was determined the microbes were digesting the collagen found in the tendon using special collagenase enzymes [37]. Many years later, it was questioned if these collagenases could prove useful for diseases such as Dupuytren’s contracture and PD. In 1982, a group of scientists pioneered CCh research by demonstrating that it could reduce the size of PD plaques without affecting surrounding tissues like skin, vessels, and nerves [29].

Almost a decade after this potential breakthrough in using CCh for fibrotic diseases, the same group completed the first randomised, double-blind, placebo-controlled study using CCh on 49 men with PD; it was reported that there was a significant difference in the plaque size of men treated with CCh when compared to the placebo [30]. The study divided men into three different groups and administered different dosages of CCh to each group. The three groups were chosen based on the degree of penile curvature; the group with the least penile deviation received the lowest dose of 0.35 mg, the middle group received 0.58 mg, and the men with the highest degree of curvature were administered the maximum dose of 0.81 mg [30]. It was found that men in the 0.58 mg treatment group reported the best outcomes, thus the 0.58 mg dose became the standard protocol of CCh injection for future studies [30,38].

Once the efficacy of CCh had been established, it was important to determine the most effective treatment regimen. How often should one receive an injection? How long should a patient be administered CCh therapy? Without a set standard, scientists explored different treatment schedules in a phase II trial using three unique IL treatments of CCh at 0.58 mg over a span of 7–10 days. Then, the treatment was repeated 90 days later, at which points progress was assessed [31]. Although no placebo was utilised, this study reported that 58% of men had a decrease in curvature, with 95% showing a decrease in plaque size [31]. Moreover, according the questionnaire following the conclusion of the study, almost all the patients indicated substantial enhancement of their sexual performance and satisfaction [31].

With CCh showing promise in combatting PD symptoms, the drug entered the Phase III Investigation for Maximal Peyronie’s Reduction Efficacy and Safety Studies (IMPRESS) trials. It consisted of two double-blinded, randomised, placebo-controlled trails that spanned 1 year [32]. Composed of >800 men, these studies observed men through a maximum of four cycles of injections, separated by 6 weeks [32]. Per cycle, the patients received two injections of 0.58 mg CCh with a subsequent ‘modelling’ (where the penis is manually stretched in an effort to physically break fibrotic plaques) session to conclude the cycle [32]. The authors found men in the treatment group saw an average 34% reduction in curvature with a mean (SD) – 17.0 (14.8)° of difference per person, while men in the placebo saw an average 18.2% decrease in penile bend with a mean (SD) – 9.3 (13.6)° of change per person (*P* < 0.001) [32]. Treatment-related adverse events were recorded in a significant number of patients but these complications such as swelling or bruising were considered unextraordinary and almost all required no medical involvement; however, there were three reports of corporal tear and three more instances of haematoma formation that required surgical intervention [32].

Since CCh was officially approved by the FDA to treat PD in 2013, the breadth of studies regarding the treatment has been lacklustre. However, researchers have begun to focus on the efficacy of CCh during the acute phase of PD. In the first report to assess the usage of CCh during the acute phase, Nguyen et al. [33] found no significant difference in degrees of curvature in patients in chronic and active phases of PD; furthermore, the study showed no significant difference in the treatment-related adverse events between the chronic and active groups. Similarly, Hellstrom et al. [34] found the phase of PD to be a poor predictor of how well a patient will respond to CCh. In a small review of current data, it was determined that CCh was effective at reducing penile curvature (27.4–37.4%) while displaying no unique adverse reactions during the acute phase [39]. The current data needs to be corroborated by a multi-institutional analysis regarding the usage of PD in the acute phase to be of more value.

It has been reported that since the advent of CCh therapy, men have been more likely to prefer IL therapy and providers have been less likely to utilise surgery as the primary solution to PD [40]. Additionally, researchers reported a novel and truncated protocol that yields comparable results to the standard procedure set forth in the IMPRESS trials. In this new approach, the number of injections per cycle was reduced to one, the dosage was increased from 0.58 to 0.9 mg, and the patients only had to make an office visit four times over a 12-week duration [35]. At the conclusion of the study, the authors found an average – 31.4% reduction in penile curvature [35]. When juxtaposed to the – 34% reduction observed in the IMPRESS trials, the shortened protocol proves to be cheaper and quicker with comparable results.

A recent systematic review of the literature (1982–2020) of all IL injection therapies for patients with atypical PD supported the expanded ‘off-label’ use of CCh in patients with atypical PD who represent 10% of the entire PD population (ventral curvature, hourglass narrowing, unilateral indentations, severely shortened penile length, and multiplanar curvatures). These patients could be excluded from receiving CCh due to combined effect of providers’ hesitation due to ‘off-label’ indication, potential significant financial burden, and risk of urethral injury, especially for ventral curvature. Only a few studies (15 out of 488) included patients with atypical PD. A total of 250 patients with atypical PD out of 1357 were treated with IL therapies, most patients 162 (64.8%) were treated with CCh, 49 (19.6%) with verapamil, 29 (11.6%) with IFN alfa-2b, five (2.0%) with HA, and five (2.0%) with onabotulinumtoxinA (Botox®; AbbVie Pharmaceuticals, Chicago, IL, USA). There were no reports of urethral injury and only one case of penile fracture requiring surgical intervention [39].

However, it is worth noting that due to a variety of economic factors, CCh is no longer commercially available anywhere outside the United States, thus potentially impacting the treatment of millions of men with PD who may have previously benefitted from it [41].

## Conclusion

Peyronie’s disease is estimated to affect 0.5–13% of men in the USA [42]. The wide range of data may be explained by varying severities and presentations of the condition and perhaps stigma and shame surrounding a man’s choice to seek medical attention. Regardless, it is known to affect a significant number of men, thus it is important adequate research is performed to develop the most effective means of ameliorating this disease. Since the approval of CCh by the FDA in 2013, it has been a staple in the treatment of PD, and here we report the continued success and superiority of this therapy.
